# Non-Linear Association of Exercise with Animal Protein Consumption among U.S. Adults

**DOI:** 10.3390/nu16101438

**Published:** 2024-05-10

**Authors:** Justin D. Bina, Glynn T. Tonsor

**Affiliations:** Department of Agricultural Economics, Kansas State University, Manhattan, KS 66506, USA; gtonsor@ksu.edu

**Keywords:** animal protein, consumption, NHANES, physical exercise, protein

## Abstract

Supply chain disruptions, human and animal health concerns, and environmental impacts of livestock production have spurred renewed attention to animal protein consumption in the U.S. Prior research has sought to better understand animal protein consumption by assessing demographic and socioeconomic determinants. However, physical exercise represents a behavioral determinant of consumption that is societally important and, thus far, has not been considered in empirical assessments. Our objective was to quantify the association of exercise with the consumption of total protein, beef, pork, poultry, seafood, eggs, and dairy among U.S. adults. We analyzed 24 h dietary recall and physical activity data from adults in the 2007–2018 National Health and Nutrition Examination Survey (NHANES). The association of exercise with protein consumption (total and disaggregated animal protein) was assessed using ordinary least squares and multivariate Tobit regression. Non-linear associations of exercise with total protein consumption were found, with the magnitude of association highest at 121–180 min per day of exercise. Non-linear associations were also found with animal protein consumption, which differed in sign and magnitude across protein sources. The magnitudes of association, paired with a sizable share of the study sample engaged in exercise, suggest a substantial influence of exercise on protein consumption habits in the U.S.

## 1. Introduction

U.S. animal protein consumption has garnered renewed attention in recent years. Coronavirus disease 2019 (COVID-19) impacted the economic markets for meat and eggs, with simultaneous supply disruptions and consumer “panic buying” pushing prices for those goods over historical highs [1,2,3]. News media covered the phenomenon for months [4,5], while economic literature devoted to supply chain performance in domestic meat markets quickly appeared in refereed journals. Similarly, and soon thereafter, the avian influenza outbreak of 2022 resulted in sizable reductions in the U.S. commercial chicken and turkey flock, again impacting the availability and prices of poultry and egg products in U.S. grocery stores [6,7,8].

Human and animal health factors have not been the only drivers of renewed interest in animal protein consumption. Sustainability in agriculture and concerns over the environment have also contributed to recent discussion surrounding livestock production and the consumption of food products derived from animals. Willits-Smith et al. [9] is a noteworthy example of these discussions, noting the environmental impacts of U.S. beef production and evaluating the demographic and behavioral determinants of disproportionate beef consumption. Additionally, plant-based meat alternatives have become a “hot topic” in the U.S. food industry, serving as a source of protein for consumers who are worried about the environmental impact of livestock production, the handling of livestock raised for human consumption, or the human health impacts of animal-derived food products. The emergence of these meat alternatives has prompted economic research on consumer demand for alternative and traditional meat products, how U.S. food consumers substitute between the two, and how demand for alternative meat impacts environmental and climate outcomes [10,11,12].

Recent attention to domestic meat markets and, more generally, animal protein consumption, warrants updated empirical assessments. A host of prior work has assessed trends and correlates of animal protein consumption [9,13,14,15,16,17,18,19] or has quantified the impact of animal protein consumption on human health outcomes [20,21,22,23]. This work has varied in the period of study, the aggregation of animal protein intake (i.e., all animal protein consumption, processed meat consumption, beef consumption, etc.), and the demographic characteristics and health outcomes of key interest. As an example, recent research has found that 12 percent of Americans were responsible for half of all beef consumed in the U.S. from 2015 to 2018, with males being more likely than females to consume a disproportionate amount of beef [9]. Similar efforts explored seafood consumption, finding that 56 percent and 31 percent of seafood purchases (by weight) occurred in retail and restaurants, respectively, from 2007 to 2016 [15]. Related to human health outcomes, high protein intakes were found to be associated with modest increases in appendicular skeletal muscle mass in non-obese and physically inactive Americans (aged 50 years or older) [22]. Further, meat consumption, specifically, was found to be positively associated with obesity among U.S. adults [23] but not with mortality [21]. Though the quantity of research dedicated to understanding animal protein consumption is sizable, there exists an important knowledge gap. Physical exercise has not been assessed as a potential determinant of total protein and disaggregated animal protein consumption We note that Kappeler et al. [21] controlled for the number of days spent in moderate or vigorous physical activity per week when estimating the association of meat intake with mortality, but did not quantify the association of physical exercise with meat or protein intake; and Wang and Beydoun [23] controlled for physical activity when examining the association of meat consumption with obesity but, again, did not assess the association of physical exercise with meat or protein intake. It was our objective to quantify the association of physical exercise with the consumption of total protein, beef, pork, poultry, seafood, eggs, and dairy among U.S. adults. The remainder of this introductory section further motivates that objective.

The National Academy of Medicine (formerly the Institute of Medicine) has provided for decades a series of dietary reference intakes that are intended to serve as a guide for good nutrition and have informed most food and nutrition policies and programs [24]. The recommended dietary allowance (RDA) is one of such reference intakes, defined as “the average daily dietary nutrient intake level that is sufficient to meet the nutrient requirements of nearly all (97–98 percent) healthy individuals in a particular life stage and gender group” [24]. For total protein, the RDA for all adults aged 19 years and over is 0.80 g per kilogram of bodyweight per day (g/kg/d) [24]. To put the National Academy of Medicine [24] recommendation into context, an 80 kg individual would need to consume 64 g of pure protein per day to meet the RDA, which equates to around 206 g of boneless, skinless, and roasted chicken breast [25].

Important to this study, and in part inspiring our objective, some prior work in the field of nutrition has noted that higher intakes of protein are needed for individuals who are physically active [26,27]. In particular, the International Society of Sports Nutrition, in their official stance on protein requirements for exercising individuals, recommend that training athletes consume in the range of 1.4 to 2.0 g/kg/d [26]. This range of intake corresponds to up to 160 g of pure protein per day for an 80 kg individual, or up to 516 g of boneless, skinless, and roasted chicken breast [25]—substantially higher intake levels than those recommended by the National Academy of Medicine [24] for the average individual.

The purpose of our research was not to determine the adequate level of protein intake for those who are engaged in physical exercise pursuits. However, we highlight that discussion surrounding the relationship between physical exercise and protein consumption has migrated from nutritional studies to popular press and social media. Examples of this discussion are abundant in print and digital news media [28,29], and can be easily found across social media platforms (e.g., TikTok, YouTube, etc.) from a simple search of the terms “protein”, “fitness”, “exercise”, etc. Additionally, and from the U.S. Bureau of Labor Statistics [30] estimates, 20.1 percent of Americans aged 15 years and over engaged in some form of sport, exercise, or recreation on an average day in 2022. This prevalence of exercise, paired with the documented societal interest in its relationship to protein, suggests important implications on nationwide health outcomes (e.g., skeletal muscle mass among older Americans, obesity prevalence, etc.) and the economic markets for protein-dense food products.

This research contributes to the general understanding of the determinants of animal protein consumption by (1) evaluating determinants across a variety of animal protein sources previously unstudied (i.e., pork, eggs, and dairy) and (2) considering a potentially important behavioral component of consumption (i.e., physical exercise). The results from this study can be used to inform food and nutrition programs focused on protein intake, educate U.S. food producers on consumer behavior in domestic protein markets, and guide future academic research on other food groups.

## 2. Materials and Methods

### 2.1. Sample

This study utilized survey data from the 2007–2008 through 2017–2018 cycles of the National Health and Nutrition Examination Survey (NHANES), which includes 75,402 participants in total. Conducted by the National Center for Health Statistics (NCHS) branch of the Centers for Disease Control and Prevention, NHANES is a nationally representative survey designed to assess the health and nutritional status of civilian, non-institutionalized adults and children in the U.S. [31]. Important to this study, demographic and socioeconomic characteristics of NHANES participants are obtained during in-home interviews [32]. Physical activity data and a 24 h dietary recall are obtained during visits to a mobile examination center (MEC) with a second, follow-up 24 h dietary recall being obtained for most participants (approximately 87 percent) via telephone between three and ten days following the MEC exam [32]. The analytical sample was restricted to participants aged 18 years and older with complete data for physical activity (*n* = 36,525). Assessments of protein consumption were further restricted to participants who provided at least one reliable 24 h dietary recall (*n* = 32,375). Further detail on survey design and data collection is provided elsewhere, along with NCHS Ethics Review Board approval of the NHANES program [33,34]. As this study analyzes publicly available data, it did not require approval from our institutional review board.

### 2.2. Dietary Intake

Dietary intake is reported in the NHANES databases as it was consumed. That is, food items and estimated quantities are reported without specifying quantities of the individual components (i.e., the quantity of beef or cheese contained within a “burrito” is not reported). While NHANES data do provide caloric, macronutrient, and micronutrient content of each reported food item, as-consumed food item amounts need to be translated to equivalents of their respective animal protein components. To address this issue, we utilized the USDA Agricultural Research Service’s Food Patterns Equivalents Database (FPED), which was developed to convert NHANES-reported food items into their corresponding ounce, cup, or gram equivalents of broad nutritional food components [35]. These food components include, among others, (1) total meat, poultry, seafood, organ meats, and cured meat; (2) eggs (chicken, duck, goose, quail) and egg substitutes; and (3) total milk, yogurt, cheese, and whey.

Combining FPED equivalents data with text from the food description for each NHANES-reported food item, we allocated equivalents to beef, pork, poultry, seafood, combination (i.e., multi-meat), egg, and dairy categories (dairy is measured in cup equivalents; meat and eggs are measured in ounce equivalents). For instance, if the description of a reported food item contained beef (e.g., “Beef, steak, flank”) and mentioned no other meat, the full amount of the “total meat, poultry, seafood, organ meats, and cured meat” ounce equivalents was allocated to beef. If the food description included beef and another meat (e.g., “Bacon cheeseburger, 1 medium patty, plain, on white bun”) or did not mention a specific meat (e.g., “Frankfurter or hot dog, plain, on bun”), the ounce equivalents were allocated to the combination meat category. It is important to note that this combination meat category primarily includes frankfurters and hot dogs, bologna, pepperoni, and other cured or luncheon meat derived from multiple animal protein sources. In the instance of multi-meat food items, some prior work allocated a fixed proportion of ounce equivalents to each meat category [9,16]. For example, half of the total meat ounce equivalents of a bacon cheeseburger would be allocated to beef and half to pork. We elected instead to utilize a combination category, as the share of multi-meat food product quantity belonging to each animal protein source is highly product-specific and cannot be determined without substantial subjectivity on behalf of the researcher. However, our utilization of a combination meat category understates the true consumption of beef, pork, poultry, and seafood.

For the 87 percent of participants who provided two reliable 24 h dietary recalls, daily intakes were calculated as an average across the two days [18,19,23]. Otherwise, daily intakes reflect only the first dietary recall obtained from the MEC exam. It should be noted that the measures of dietary intake for protein and disaggregated animal proteins utilized in this study do not reflect participants’ usual intakes. For episodically consumed foods such as beef or seafood, our dietary intake construction does not correct for measurement error inherent in one- or two-day food consumption recalls and overestimates the true number of non-consumers. Some NHANES-based consumption studies have implemented the National Cancer Institute (NCI) method of calculating usual intakes that are more representative of individuals’ typical food consumption patterns [16,17,22]. However, we elected to analyze raw, rather than usual, intakes for two primary reasons: (1) the NCI method does not allow for predicted usual intake values of zero and (2) measurement error in the outcome variable does not bias estimates of the association of predictor with outcome if the measurement error is random and independent of the predictors ([36], pp. 76–78).

Regarding the former, we do not expect all participants to be consumers of disaggregated animal proteins such as seafood and, as such, elected against a method that does not allow for non-consumption. Further, statistical methods have been developed to account for censored data (i.e., non-consumption) and provide important insights into marginal effects of predictors on uncensored and censored outcomes. We discuss one such method in ensuing sections. Regarding the latter, as our primary objective was to quantify the association of physical exercise with animal protein consumption, measurement error in the outcome variable (i.e., animal protein intake) is not overly important because it does not bias estimates of that association. We do, however, report the number of non-consumers in the descriptive statistics for each animal protein source, as statistics on raw intake are influenced by measurement error.

### 2.3. Physical Exercise

NHANES participants are asked a sequence of questions regarding their physical activity. First, participants are asked, “In a typical week {do you/does SP} do any moderate-intensity [vigorous-intensity] sports, fitness, or recreational activities that cause a small [large] increase in breathing or heart rate such as brisk walking, bicycling, swimming, or volleyball for at least 10 min continuously?” If answering “yes” to this question, participants are then asked, “In a typical week, on how many days {do you/does SP} do moderate-intensity [vigorous-intensity] sports, fitness or recreational activities?” and “How much time {do you/does SP} spend doing moderate-intensity [vigorous-intensity] sports, fitness or recreational activities on a typical day?”.

Moderate and vigorous physical activity was coded as zero minutes per day (min/d) if a participant answered “no” to the first question. Conditional on having answered “yes” to the first question, participants’ daily moderate and vigorous physical activity was then calculated by multiplying the number of days per week they partake in moderate and vigorous activity and the minutes per day spent in that activity on those days, all divided by seven days per week to obtain a daily average. We combined daily moderate and vigorous activity into one aggregate physical exercise variable that is of key interest in the remainder of this study.

### 2.4. Statistical Analysis

Weighted means of daily physical exercise were computed by survey cycle, demographic characteristic, and body weight. Further, the concentration of exercise by demographic cohort across the entire period of study was computed by dividing each cohort’s total minutes of daily physical exercise by the grand total of daily physical exercise reported by all NHANES participants. These analyses incorporated the MEC exam sample weights, which were adjusted to account for the combination of six NHANES survey cycles (2007–2008 to 2017–2018), following NCHS [37] suggested methods. Weighted means of aggregate protein consumption and disaggregated animal protein consumption were also computed by survey cycle, demographic characteristic, body weight, and physical exercise level using the dietary day one sample weight suggested by NCHS [37], again adjusted for the combination of survey cycles. Dietary day one sample weights were likewise utilized in the regression analyses discussed below.

The association of daily physical exercise with total protein consumption was assessed through univariate ordinary least squares (OLS) regression. Since protein consumption is never zero, methods accounting for censored (i.e., zero) observations were not necessary. Further, total protein consumption was not estimated jointly with disaggregated animal protein consumption because increases or decreases in animal protein consumption necessarily increase or decrease total protein consumption. All regression analyses utilized categorical physical exercise variables as there were a substantial number of non-active (i.e., zero minutes of daily physical exercise) NHANES participants that may have substantially different protein consumption. Additionally, categorical (versus continuous) physical exercise variables allow for non-linearity in the association of exercise with protein consumption. Finally, we included controls for survey cycle (capturing temporal differences in consumption), age, gender, race, educational attainment, and annual household income, which may confound the association between protein consumption and physical exercise if omitted.

We did not, however, control for body weight as it is determined with protein consumption simultaneously, which can yield biased parameter estimates unless appropriate instrumental variables can be identified and utilized in estimation. Wooldridge ([36], pp. 89–114) provides further motivation for instrumental variables estimation of single-equation linear models. We do note, however, that omitted variable bias exists by not having controlled for body weight. Since body weight is positively correlated with both the outcome (i.e., intake, consumption) and predictor (i.e., physical exercise), estimates of the association of exercise with consumption may be biased upward. Justifying the omission of body weight as a covariate, and providing confidence in our final results, a model specification that did control for body weight yielded OLS estimates that were not appreciably different than the primary specification we present.

To account for the censored nature of episodically consumed animal proteins, the association of physical exercise with these intakes was assessed through censored, or Tobit, regression [38,39]. Further, animal proteins were estimated jointly in a multivariate Tobit model to account for interdependencies in the economic markets for each of these protein sources and the corresponding correlation between errors in each equation. The multivariate Tobit model was estimated via maximum likelihood estimation, again incorporating physical exercise as a series of categorical variables and controlling for survey cycle, age, gender, race, educational attainment, and annual household income.

Given the substantial number of non-consumers for the respective animal proteins, latent marginal effects from the multivariate Tobit are not overly meaningful. As such, we computed marginal effects on the expected value for intake for all observations (censored and uncensored) and for uncensored observations, which are referred to as unconditional and conditional marginal effects, respectively. Calculation of Tobit marginal effects can be found elsewhere [36,38] (pp. 671–676). Standard errors obtained from the original multivariate Tobit estimation do not apply to unconditional and conditional marginal effects. To obtain appropriate standard errors, we employed a Krinsky–Robb simulation procedure. Briefly, the Krinsky–Robb method involves taking *n* draws from a multivariate normal distribution parameterized by mean being the parameter estimate vector and variance being the variance–covariance matrix obtained from the original model estimation [40]. Thus, *n* simulated parameter estimate vectors are constructed with marginal effects calculated from each, generating an empirical distribution [40]. The Krinsky–Robb procedure of calculating standard errors was utilized, rather than a bootstrap method, as it vastly reduces the computational burden of estimating *n* seven-equation multivariate Tobit models.

Data preparation and computation of weighted means were conducted using R software version 4.2.2 and the R “survey” package. OLS and multivariate Tobit estimation were conducted using SAS 9.4’s REG and QLIM procedures, respectively.

## 3. Results

Descriptive statistics, regression estimates, and marginal effects for aggregate protein, meat, and egg consumption are presented in grams per day (g/d), while dairy is presented in cups per day (c/d).

### 3.1. Means of Physical Exercise

Table 1 displays the average daily physical exercise by NHANES survey cycle and participant characteristics. The averages depicted in Table 1 are unconditional, meaning that they were computed incorporating both those individuals who reported physical exercise and those who did not. The share of non-exercising individuals (i.e., those who reported zero minutes per day in moderate or vigorous recreational activity) ranged from 0.52 in the 2013–2014 NHANES cycle to 0.58 in the 2007–2008 cycle.

The grand mean of daily physical exercise across all survey cycles and individual characteristics was 22.83 min/d. Temporal differences existed in daily physical exercise, being lowest in the 2007–2008 survey cycle at 21.03 min/day and highest in the 2011–2012 cycle at 24.70 min/day. Though declining by approximately one min/day following the 2011–2012 period, the average daily physical exercise was still 12.33 percent higher in 2017–2018 than ten years prior, highlighting a U.S. population that has become generally more physically active over time. Important to note is that substantial changes in physical exercise may have occurred following the COVID-19 pandemic; however, 2021–2023 NHANES survey data had not yet been released for analysis at the time of writing. Though not a perfect comparison to NHANES-based exercise measures, estimates from the American Time Use Survey indicate that U.S. citizens aged 15 years and over participated in 17.40 min/d of sports, exercise, and recreation in 2022, which was the same activity level reported pre-pandemic in 2018 [30,41].

U.S. males spent more time in physical exercise per day than females on average across the period of study. As of 2017–2018, males spend 28.13 min/day exercising compared to females’ 19.43 min/d—44.77 percent higher. Differences also existed by age, with daily physical exercise skewed toward younger individuals. In 2017–2018, individuals aged 18–24, 25–34, 35–44, 45–54, 55–64, and over 65 years reported daily physical exercise of 40.78, 30.83, 22.68, 18.48, 18.06, and 16.56 min/day, respectively, on average. Across all age cohorts, more time was spent in daily physical exercise in 2017–2018 than in 2007–2008. Percentage increases in daily exercise over those ten years ranged from 2.77 for those aged 45–54 years to 25.10 for those aged 65 and older. Of note is that individuals aged 34 years and younger reported daily physical exercise nearly 20 percent higher in 2017–2018 than in 2007–2008. With younger people exercising increasingly more over time, exercise pursuits may have progressively larger impacts on U.S. food consumption and in other industries (i.e., apparel and textiles, sports equipment, etc.) that cater to the exercising sub-population. This is especially true if young, exercising individuals maintain their exercise habits into later years of life, though long-running panel data is required for assessments of that phenomenon.

The time spent in exercise was not appreciably different by race, with non-Hispanic whites, non-Hispanic blacks, Mexican Americans, and all other races reporting in 2017–2018 physical exercise of 23.30, 24.32, 25.69, and 23.28 min/d, respectively, on average. Larger differences in daily physical exercise existed by individuals’ educational attainment and annual household income. Those without high school degrees, with high school degrees or some college, and having graduated college reported physical exercise of 15.06, 22.72, and 28.82 min/day on average in 2017–2018, respectively. These exercise levels reflect percentage increases of 18.91, 6.88, and 3.83 over the 2007–2008 averages. Similar trends were present in the time spent exercising by income. Individuals with annual household incomes under $20,000, between $20,000 and $74,999, and $75,000 or greater reported physical exercise of 21.08, 21.74, and 26.76 min/day on average in 2017–2018, respectively. These reflect percentage increases over 2007–2008 averages of 50.08, 7.67, and 3.06. We are unsure why individuals with less formal schooling and lower incomes reported greater relative increases in physical exercise over time. These results suggest that exercise pursuits have become less costly and more accessible over time; however, NHANES data do not allow us to further distinguish between participants’ type of exercise or its financial burden.

### 3.2. Concentration of Exercise

Table 2 depicts the concentration of daily physical exercise by demographic cohort over the entire period of study, providing an insight into which groups have disproportionate exercise activity and potentially elevated perceived protein needs. With 432 distinct cohorts (across two genders, six age groups, four races, three education levels, and three household income groupings), we display only the 28 cohorts that accounted for at least one percent of the total reported physical exercise. In total, the 36,525 NHANES participants providing exercise data accounted for 833,991 min of daily physical exercise.

White males and females aged 45–54 years having graduated from college and with at least $75,000 in annual household income accounted for the largest share of total time spent in physical exercise. These cohorts, accounting for 0.56 (male) and 0.59 (female) percent of NHANES respondents, were responsible for 2.29 (male) and 2.28 (female) percent of all reported daily physical exercise. Across the 28 cohorts accounting for at least a one percent share of total exercise, 18 were male cohorts, all were non-Hispanic white, all had at least a high school degree, and all had at least $20,000 in annual household income. These 28 demographic cohorts comprised 19.40 percent of NHANES participants used in this assessment (7086 of 36,525) but accounted for 43.31 percent of the time exercising.

### 3.3. Means of Consumption

Moving into dietary intake, the grand mean of total protein consumption across all survey cycles and individuals was 81.97 g/d, which is comparable to prior work using NHANES dietary recall data [13,17]. Further, average protein consumption per kilogram of body weight was 1.03 g/d. Among all NHANES participants, 63.24 percent met the National Academy of Medicine [24] RDA of 0.80 g/kg/d. Additionally, among just participants who reported spending some time each day in physical exercise, only 21.70 percent consumed at least the 1.40 g/kg/d of protein recommended for exercising individuals by the International Society of Sports Nutrition [26]. The grand means of beef, pork, poultry, seafood, combination meat, egg, and dairy consumption were 32.89 g/d, 19.46 g/d, 49.08 g/d, 17.64 g/d, 14.53 g/d, 12.37 g/d, and 1.22 c/d, respectively. Though differences in the period of study and disaggregation of animal proteins make comparisons to prior work difficult, the mean intakes for seafood and poultry are broadly consistent with previous findings [14,15,19], as is the relative ordering of red meat (i.e., beef and pork), poultry, and seafood [14,19].

The total number of non-consumers by NHANES survey cycle is depicted in Table 3, along with the share of participants being non-consumers. Detailed reporting of protein consumption and disaggregated animal protein consumption by survey cycle and individual characteristics is then available in Table 4.

Over the period of study, 51, 56, 35, 71, 58, 58, and 11 percent of individuals were non-consumers of beef, pork, poultry, seafood, combination meat, eggs, and dairy, respectively. Again, these descriptive statistics are influenced by our use of raw intakes for episodically consumed foods, rather than usual intakes. However, there are noticeable temporal differences, which should not be influenced by the measure of intake. Notably, the share of individuals who did not consume beef increased from 44 percent in 2007–2008 to 57 percent in 2017–2018. The share of non-consumers of pork and dairy also increased over time, though to a lesser extent. Conversely, the share of individuals who did not consume combination meats or eggs slightly decreased from 2007–2008 to 2017–2018.

Noting again the increasing share of individuals who did not consume beef, pork, or dairy, the average consumption level of these animal proteins decreased from 2007–2008 to 2017–2018. Pork consumption decreased by 7.69 percent over the period, while beef and dairy each decreased by over 18 percent. By contrast, combination meat and egg consumption increased by 20.11 and 30.32 percent over the same time. The beef consumption decline and egg consumption growth from 2007 through 2018 are consistent with USDA Economic Research Service estimates of nationwide disappearance [42]. Beyond these simple temporal differences, substantial heterogeneity exists in consumption between demographic groups. Across all survey cycles, U.S. males reported total protein, beef, pork, poultry, seafood, combination meat, egg, and dairy consumption of 96.26 g/d, 43.12 g/d, 25.16 g/d, 56.61 g/d, 19.42 g/d, 18.63 g/d, 14.27 g/d, and 1.38 c/d, respectively, on average. These values reflect consumption that is 40.25, 84.76, 78.01, 34.66, 21.51, 74.21, 34.64, and 28.26 percent higher than that of females. Gender differences in total protein and animal protein consumption found here have been consistently documented in prior assessments using NHANES dietary recall data [14,15,17,18,19]

Individuals aged 65 years and over consumed lower amounts of total protein, beef, poultry, combination meat, and dairy than younger individuals. This age cohort reported total protein consumption of 71.39 g/d, beef of 28.34 g/d, poultry of 35.39 g/d, combination meat of 10.47 g/d, and dairy of 1.12 c/d on average. By comparison, those aged 18 to 24 years consumed 82.23 g/d of total protein, 33.71 g/d of beef, 58.11 g/d of poultry, 16.32 g/d of combination meat, and 1.33 c/d of dairy on average. Prior studies have also noted relatively lower total protein, red meat, and poultry consumption for the elderly sub-population [13,14,18,19]. Mexican Americans averaged the highest levels of total protein, beef, combination meat, and egg consumption at 86.86 g/d, 36.35 g/d, 19.15 g/d, and 17.44 g/d, respectively. Beasley et al. [13] likewise found higher total protein consumption among Hispanics, while Daniel et al. [14] and Zeng et al. [19] similarly found higher consumption of unprocessed red meat. Conversely, non-Hispanic blacks reported the highest average consumption of poultry and seafood at 64.54 g/d and 22.54 g/d, respectively, which is broadly consistent with the findings of Daniel et al. [14] and Zeng et al. [19]. Non-Hispanic whites accounted for relatively higher rates of pork and dairy consumption at 20.07 g/d and 1.35 c/d, respectively.

Differences also exist in consumption by individuals’ educational attainment and annual household income for most protein sources. Those having graduated from college reported relatively higher consumption of poultry (51.57 g/d), seafood (20.67 g/d), and dairy (1.29 c/d), and relatively lower consumption of beef (28.62 g/d), pork (16.82 g/d), and combination meat (11.75 g/d). Similarly, individuals in the highest income bracket consumed poultry (52.64 g/d), seafood (20.03 g/d), and dairy (1.29 c/d) at higher rates than lower earners. Regarding total protein consumption, college graduates and those with annual household incomes of at least $75,000 consumed 85.80 and 86.27 g/d, respectively, while those without a high school degree or earning less than $20,000 annually consumed around 77 g/d in total protein on average.

Interestingly, individuals who do not partake in physical exercise had substantially lower daily total protein consumption at 77.68 g/d but did not always have a lower average consumption of specific animal proteins. This indicates that those who exercise—generally consuming more total protein each day than those who do not—may be disproportionately seeking protein from sources that are not derived from animals or do not find some animal sources of protein to be sufficient for their health- and fitness-related needs. Excluding daily physical exercise categories with fewer than 500 respondents (i.e., 181–240 min/d, 241–300 min/d, and greater than 300 min/d), non-exercisers had the lowest average consumption of total protein (77.68 g/d), poultry (44.98 g/d), seafood (16.30 g/d), and dairy (1.14 c/d). Conversely, the 571 NHANES participants that reported daily physical exercise of 121 to 180 min had the highest average consumption of total protein (98.55 g/d), beef (39.21 g/d), poultry (67.38 g/d), seafood (23.43 g/d), eggs (15.24 g/d), and dairy (1.51 g/d). Important to note is that powders or liquid concentrates that are meant to be added to liquids (i.e., protein powders) are included in NHANES prior day intake reporting, classified as “beverages” [43]. Thus, dietary supplements such as whey protein powders are included in our assessment and are reflected in estimates of dairy consumption. This explains in part the higher reported dairy consumption among heavy exercisers and lower dairy consumption among those who do not exercise.

### 3.4. Association between Physical Exercise and Protein Consumption

The results of the total protein OLS regression are presented in Table 5. Estimates with *p*-values of 0.05 or lower were considered to be statistically significant. The default values for the categorical variables are 0 min/d of physical exercise, the 2007–2008 NHANES survey cycle, females, age 18 to 24, non-Hispanic white, no high school degree, and annual household income less than $20,000. An additional categorical variable was included for NHANES participants who did not provide data on annual household income (*n* = 1705) to determine if these individuals were statistically different in their protein consumption and to avoid the omission of a sizable portion of the study sample.

Physical exercise was positively associated with total protein consumption up to levels of 240 min/d (four hours). Exercising one half hour or less per day was associated with an additional 2.08 g/d of protein consumption relative to not exercising. This reflects total protein consumption that is 3.32 percent higher than that of non-exercisers, holding all other predictors at their default values. The magnitude of association was greater for higher levels of daily exercise, peaking at an additional 12.41 g/d of protein consumption when exercising between two and three hours per day (relative to not exercising), or 19.81 percent higher protein consumption when holding all other predictors at their default values. Physical exercise exceeding 240 min/d was not associated with elevated total protein consumption at the 95 percent confidence level. As only 132 NHANES participants reported physical activity exceeding 240 min/d, this result could be driven by outliers within the small sub-sample. Alternatively, individuals participating in this level of physical exercise may be engaged in recreational pursuits that are not intended to build muscle (e.g., distance running) and do not have a perceived elevated need for protein. Again, we cannot distinguish the type of physical exercise using NHANES data.

Derived from a multivariate Tobit regression, conditional marginal effects on disaggregated animal protein consumption are depicted in Table 6. These reflect the effects of the predictors on uncensored observations (i.e., the marginal effects on animal protein consumption conditional on consumption being non-zero). We forego detailed discussion on unconditional marginal effects but include those estimates in Appendix A Table A1.

The association of physical exercise with animal protein consumption differed by sign and by protein source. Exercise levels of 31 to 60 min/d and 61 to 120 min/d were associated with reductions in daily beef consumption of 13.30 g and 16.13 g, respectively, relative to not exercising. Pork and combination meat also displayed a negative association with physical exercise, with magnitudes of association being highest at 181 to 240 min/d of physical exercise for pork (marginal effect of −19.30) and 61 to 120 min/d of physical exercise for combination meats (marginal effect of −11.32). Consider that, among those who reported non-zero consumption, the mean consumption was 66.87 g/d, 44.37 g/d, and 32.77 g/d for beef, pork, and combination meat, respectively. The negative marginal effects of physical exercise between 31 and 120 min/d witnessed for each of these protein sources represent 20–24, 17–19, and 31–35 percent reductions from the conditional means, respectively. These results indicate that, although individuals who partake in physical exercise consume more total protein than those who do not, some protein sources are less desired. This may be due to a nutritional content of beef, pork, and combination meat products (e.g., luncheon meat) that does not align with exercisers’ perceived nutrition needs.

Conversely, physical exercise generally had a positive association with poultry, seafood, egg, and dairy consumption. The magnitude of association was largest for poultry at 181 to 240 min/d of exercise (marginal effect of 27.88), for seafood at 31 to 60 min/d (marginal effect of 26.46), for eggs at 181 to 240 min/d (marginal effect of 13.01), and for dairy at 121 to 180 min/d (marginal effect of 0.60). The conditional mean consumption for poultry, seafood, eggs, and dairy was 75.62 g/d, 65.14 g/d, 30.52 g/d, and 1.34 c/d, respectively. Looking again at exercise levels between 31 and 120 min/d, the marginal effects represent 14–18, 36–41, 13–29, and 7–30 percent increases from the conditional means for poultry, seafood, eggs, and dairy, respectively. These effects suggest a substantially higher consumption of these protein sources for individuals who exercise between one half hour and two hours per day. As these individuals make up 19.95 percent of the study sample (6460 of 32,375 participants with a reliable dietary recall) in a nationally representative survey, consumption differences among this behavioral group can have a notable influence on nationwide health outcomes and the economic markets for various protein sources.

## 4. Discussion

Prior research efforts have been conducted to determine trends and correlates of total protein and animal protein consumption in the U.S. Though differing in outcome measure and individual characteristics of key interest, our results are consistent with the conclusions of previous studies regarding consumption by gender, age, and race/ethnicity. To complement that literature, this study used NHANES data from 2007 to 2018 to assess determinants of consumption across a broader range of animal protein sources (i.e., pork, eggs, and dairy) and, importantly, considered physical exercise habits, which have thus far been an understudied behavioral component of U.S. food consumption.

A host of conclusions were drawn from our results. First, from descriptive statistics for physical exercise, over half of the U.S. population was not engaged in physical exercise over the period of study. However, the average time spent in physical exercise per day generally increased over the ten-year period, being roughly 24 min/d in 2017–2018. Roughly 20 percent increases in physical exercise from 2007–2008 to 2017–2018 were reported among citizens aged 34 years and younger, suggesting that—to the extent this trend continues and younger people maintain their exercise habits into later years of life—exercise pursuits will have progressively larger impacts on U.S. food consumption and the broader economy. Further, those with the least education and annual household income reported the greatest relative increases in daily physical exercise from 2007–2008 to 2017–2018. Physical exercise may have become less costly, or otherwise more accessible, for these demographic groups over time. However, the nature of NHANES physical activity reporting precludes us from determining the type or cost of participants’ exercise pursuits. Finally, non-Hispanic white participants with at least a high school degree and having at least $20,000 in annual household income accounted for a disproportionate share of the total minutes spent in exercise per day.

Physical exercise was positively associated with total protein consumption up to the level of 240 min/d. The magnitude of association ranged from an additional 2.08 g/d (1 to 30 min/d of exercise) to 12.41 g/d (121 to 180 min/d of exercise), relative to the levels of non-exercisers. Physical exercise generally exhibited a negative association with consumption of beef, pork, and combination meats. Conversely, a positive association was observed with consumption of poultry, seafood, eggs, and dairy. Further, the marginal effects of physical exercise on disaggregated animal protein consumption were sizable in magnitude (see Table 6) and suggest substantial differences in protein consumption patterns between those who exercise and those who do not. Though prior research has not evaluated the association of physical exercise with animal protein consumption in a national or observational setting, these results confirm what has been observed at least anecdotally in popular press and social media: not all protein sources are equal in the eyes of those who exercise. Differences in nutritional content, relative costs, and convenience likely influence which animal protein sources are most consumed by the exercising behavioral group. Red meat or further processed (i.e., smoked, cured) products may be less appealing to the exercise- and fitness-conscious population due to potential concerns over dietary cholesterol, sodium, other nutritional considerations, or price. On the other hand, animal protein sources with a lower fat content (e.g., chicken, fish), being less costly (e.g., eggs), or being more convenient (e.g., dairy-based powders) may be more desired by this behavioral group. It is important to remember, however, that nutritional content, price, and convenience of consumption varies within an animal protein source. This means that, as an example, avid exercisers may desire to consume less 73 percent lean ground beef and more 93 percent lean ground beef relative to those who do not exercise. We encourage those finer, product-specific consumption patterns to be assessed in future work.

A limitation of this work is that it was based on only the first day of dietary recall conducted in the NHANES. However, the average daily protein consumption depicted in Table 4 is largely consistent with prior refereed work that has utilized the raw dietary intake in various ways. Further, our primary objective of quantifying the association of physical exercise with consumption of total protein, beef, pork, poultry, seafood, eggs, and dairy among U.S. adults is not impacted by a potentially mismeasured outcome variable ([36], pp. 76–78). Additional limitations are that (1) we used NHANES data up to only the 2017–2018 survey cycle and (2) we could not distinguish between types of physical exercise. Regarding the former, NHANES data collection efforts were disrupted during COVID-19. Data collection and analysis for a 2021–2022 sample was expected to occur through 2023, with the data being released to the public in mid-to-late 2023 [44]. However, at the time of writing this study in 2024, that data had not yet been made available. Regarding the latter, it is likely that differences in protein consumption exist among those who engage in different exercise pursuits. For example, an avid weightlifter may desire more protein for the purpose of aiding muscle growth. Unfortunately, NHANES reporting only captures aggregate time spent in physical activity and, as such, we cannot speak to the more intricate details of its association with protein consumption.

The marginal effects found in this study, the NHANES and Bureau of Labor Statistics [30] estimates of exercise prevalence, and the recent findings that over 30 percent of the U.S. adult population intentionally consumes protein to aid in their fitness-related goals [45] suggest large and meaningful influences of physical exercise on the U.S. food system. This has several implications. First, any form of intervention to address animal protein consumption needs to consider consumers’ behavioral characteristics—importantly, their physical exercise habits. This mirrors comments made by Willits-Smith et al. [9] in their discussion of methods to reduce the consumption of environmentally impactful foods. As an ancillary point and related to Willits-Smith et al. [9], our results showed that physical exercise has a negative association with consumption of beef—a source of animal protein that has a relatively higher impact on aggregate greenhouse gas emissions. Higher levels of physical exercise among the U.S. population may reduce demand for beef, eventually resulting in cattle inventory reductions and reduced emissions in that sector. Second, U.S. livestock producers and food manufacturers need to consider more seriously the exercising behavioral group. Representing a sizable share of the market for protein and demonstrating significant differences in protein consumption, this group may substantially drive profit potential for producers in the U.S. food sector. To take advantage of these differences in consumption and capture additional revenue, food pricing and marketing strategies should be tailored to the exercising population. Additionally, changes in the prevalence of physical exercise may substantially alter the demand for animal-derived protein goods, which, in turn, should inform livestock production decisions. Finally, and on a related note, the economic markets for animal protein are susceptible to the preferences and purchasing behavior of those engaged in physical exercise. This has price and economic welfare implications for all U.S. consumers of animal protein, which future research efforts can empirically assess. It is our hope that this study motivates those future assessments.

## 5. Conclusions

In conclusion, we used a nationally representative U.S. sample to quantify the association of physical exercise with consumption of total protein, beef, pork, poultry, seafood, eggs, and dairy. Roughly half of U.S. adults were engaged in some type of physical exercise from 2007 to 2018, with the average time spent in physical exercise increasing from 21 min/d in 2007–2008 to 24 min/d in 2017–2018. Nineteen percent of the study sample accounted for 43 percent of time spent in physical exercise, with this subsample comprised of non-Hispanic white individuals with at least a high school degree and at least $20,000 in annual household income. Non-linear associations of physical exercise with total protein consumption and disaggregated animal protein consumption were present. Physical exercise displayed a positive association with total protein, poultry, seafood, eggs, and dairy consumption. Conversely, physical exercise displayed a negative association with consumption of beef, pork, and combination meats. This research can be used to inform healthy eating campaigns, improve marketing efforts and profit potential among U.S. livestock and food producers, and better understand the social welfare impacts that the exercising behavioral group has in the U.S. food industry.

## Figures and Tables

**Table 1:** Daily physical exercise by NHANES survey cycle and participant characteristics.

(a)						
	Daily Physical Exercise (min/d)					
	2007–2008 (*n* = 6216)		2009–2010 (*n* = 6521)		2011–2012 (*n* = 5858)	
	Mean (SE)	*n*	Mean (SE)	*n*	Mean (SE)	*n*
Survey Cycle						
2007–2008	21.03 (0.59)	6216				
2009–2010			21.40 (0.57)	6521		
2011–2012					24.70 (0.73)	5858
2013–2014						
2015–2016						
2017–2018						
Gender						
Male	25.42 (0.98)	3058	26.36 (0.90)	3170	29.34 (1.17)	2893
Female	16.93 (0.69)	3158	16.78 (0.71)	3351	20.37 (0.88)	2965
Age						
18–24	34.06 (1.97)	749	32.99 (1.94)	846	46.03 (2.83)	846
25–34	26.02 (1.62)	923	24.53 (1.50)	1012	27.43 (1.56)	947
35–44	22.06 (1.38)	994	22.19 (1.32)	1090	24.42 (1.71)	926
45–54	16.54 (1.17)	1000	19.09 (1.27)	1072	21.54 (1.62)	939
55–64	17.30 (1.62)	995	17.73 (1.42)	978	17.94 (1.51)	951
65+	13.23 (0.93)	1555	14.88 (0.95)	1523	16.03 (1.39)	1249
Race/Ethnicity						
Non-Hispanic White	22.00 (0.78)	2849	22.68 (0.76)	3077	25.28 (1.01)	2105
Non-Hispanic Black	19.07 (1.19)	1299	19.88 (1.30)	1193	24.88 (1.29)	1553
Mexican American	17.63 (1.27)	1097	16.25 (1.14)	1224	20.46 (2.01)	589
Other	19.58 (1.57)	971	19.47 (1.34)	1027	24.25 (1.43)	1611
Education *						
College Graduate	27.75 (1.37)	1100	28.30 (1.16)	1259	30.05 (1.37)	1397
High School/Some College	21.26 (0.82)	3125	20.72 (0.77)	3356	24.40 (1.02)	3016
<High School	12.67 (0.91)	1976	13.82 (1.18)	1889	16.13 (1.45)	1431
Annual Household Income *						
<$20,000	14.05 (1.07)	1416	16.78 (1.21)	1387	24.23 (1.78)	1429
$20,000–$74,999	20.19 (0.80)	3263	19.11 (0.82)	3362	21.98 (1.01)	2728
$75,000+	25.97 (1.25)	1284	26.88 (1.02)	1426	28.28 (1.32)	1379
Body Weight *						
50 kg or less	19.50 (3.61)	175	16.92 (3.34)	186	12.64 (1.78)	195
50.01–65 kg	20.59 (1.20)	1172	21.25 (1.36)	1203	27.30 (1.75)	1159
65.01–80 kg	22.57 (1.13)	1870	21.59 (0.94)	1990	27.21 (1.34)	1728
80.01–95 kg	23.54 (1.31)	1432	23.12 (1.24)	1526	24.83 (1.45)	1325
>95 kg	17.53 (1.17)	1233	20.06 (1.17)	1384	20.62 (1.53)	1126
**(b)**						
	**Daily Physical Exercise (min/d)**					
	**2013–2014 (*n* = 6111)**		**2015–2016 (*n* = 5973)**		**2017–2018 (*n* = 5846)**	
	**Mean (SE)**	***n***	**Mean (SE)**	***n***	**Mean (SE)**	***n***
Survey Cycle						
2007–2008						
2009–2010						
2011–2012						
2013–2014	22.33 (0.61)	6111				
2015–2016			23.73 (0.69)	5973		
2017–2018					23.62 (0.78)	5846
Gender						
Male	26.53 (1.02)	2915	27.05 (1.08)	2876	28.13 (1.23)	2834
Female	18.43 (0.68)	3196	20.65 (0.88)	3097	19.43 (0.97)	3012
Age						
18–24	34.21 (2.00)	855	36.9 (2.63)	713	40.78 (2.80)	690
25–34	28.28 (1.66)	971	28.81 (1.45)	1034	30.83 (2.02)	864
35–44	20.67 (1.32)	1021	24.31 (1.70)	946	22.68 (1.72)	823
45–54	20.58 (1.39)	963	22.32 (1.70)	953	18.48 (1.62)	834
55–64	18.10 (1.47)	996	18.19 (1.85)	956	18.06 (2.02)	1137
65+	15.23 (1.10)	1305	17.13 (1.18)	1371	16.56 (1.23)	1498
Race/Ethnicity						
Non-Hispanic White	21.82 (0.81)	2570	24.16 (1.01)	1906	23.30 (1.12)	2031
Non-Hispanic Black	23.43 (1.39)	1254	26.74 (1.45)	1260	24.32 (1.53)	1339
Mexican American	22.74 (2.00)	848	21.40 (1.39)	1061	25.69 (2.09)	789
Other	23.55 (1.20)	1439	21.22 (0.99)	1746	23.28 (1.39)	1687
Education *						
College Graduate	27.21 (1.08)	1443	29.96 (1.32)	1421	28.82 (1.64)	1336
High School/Some College	21.45 (0.87)	3284	22.99 (0.96)	3098	22.72 (0.98)	3312
<High School	16.57 (1.22)	1372	13.23 (1.16)	1446	15.06 (1.57)	1182
Annual Household Income *						
<$20,000	18.08 (1.35)	1231	20.46 (2.02)	1176	21.08 (1.87)	1008
$20,000–$74,999	21.37 (0.91)	2985	20.16 (0.86)	2846	21.74 (1.20)	2728
$75,000+	25.47 (1.06)	1602	28.78 (1.30)	1497	26.76 (1.33)	1496
Body Weight *						
50 kg or less	21.69 (3.88)	227	26.61 (4.19)	193	24.83 (9.21)	184
50.01–65 kg	25.14 (1.31)	1179	26.49 (1.82)	1121	25.96 (1.94)	1036
65.01–80 kg	23.33 (1.10)	1828	26.46 (1.27)	1732	24.59 (1.25)	1651
80.01–95 kg	22.79 (1.30)	1371	23.83 (1.57)	1347	23.78 (1.49)	1236
>95 kg	19.10 (1.25)	1253	18.45 (1.03)	1256	21.21 (1.55)	1327

Note: * Across the entire period of study, 82, 2282, and 1880 NHANES participants did not provide educational attainment, annual household income, and body weight, respectively. Observations for these traits will not match the total number of observations.

**Table 2:** Concentration of daily physical exercise by cohort.

Gender	Age	Race/Ethnicity	Education	Annual Household Income	*n*	Physical Exercise (min/d)	% of Total
Male	45–54	Non-Hispanic White	College Graduate	$75,000+	206	19,061.72	2.29
Female	45–54	Non-Hispanic White	College Graduate	$75,000+	215	19,010.09	2.28
Male	25–34	Non-Hispanic White	High School/Some College	$20,000–$74,999	370	18,896.45	2.27
Male	18–24	Non-Hispanic White	High School/Some College	$20,000–$74,999	228	17,399.66	2.09
Male	35–44	Non-Hispanic White	College Graduate	$75,000+	192	17,048.27	2.04
Female	35–44	Non-Hispanic White	College Graduate	$75,000+	243	16,498.80	1.98
Male	18–24	Non-Hispanic White	High School/Some College	$75,000+	174	14,824.28	1.78
Male	65+	Non-Hispanic White	College Graduate	$75,000+	258	14,545.52	1.74
Female	65+	Non-Hispanic White	High School/Some College	$20,000–$74,999	794	14,423.30	1.73
Female	25–34	Non-Hispanic White	College Graduate	$75,000+	209	14,402.72	1.73
Male	55–64	Non-Hispanic White	College Graduate	$75,000+	199	14,278.10	1.71
Female	18–24	Non-Hispanic White	High School/Some College	$20,000–$74,999	265	13,229.70	1.59
Male	25–34	Non-Hispanic White	College Graduate	$75,000+	160	13,202.82	1.58
Male	45–54	Non-Hispanic White	High School/Some College	$75,000+	193	12,340.75	1.48
Male	65+	Non-Hispanic White	High School/Some College	$20,000–$74,999	715	12,290.15	1.47
Female	55–64	Non-Hispanic White	College Graduate	$75,000+	142	12,084.43	1.45
Male	35–44	Non-Hispanic White	High School/Some College	$20,000–$74,999	310	11,359.87	1.36
Male	65+	Non-Hispanic White	College Graduate	$20,000–$74,999	300	11,241.49	1.35
Male	55–64	Non-Hispanic White	High School/Some College	$75,000+	171	11,067.85	1.33
Male	25–34	Non-Hispanic White	High School/Some College	$75,000+	127	10,730.18	1.29
Female	25–34	Non-Hispanic White	High School/Some College	$20,000–$74,999	321	9780.40	1.17
Male	35–44	Non-Hispanic White	High School/Some College	$75,000+	174	9597.69	1.15
Female	45–54	Non-Hispanic White	High School/Some College	$75,000+	175	9507.13	1.14
Male	55–64	Non-Hispanic White	High School/Some College	$20,000–$74,999	267	9301.67	1.12
Female	18–24	Non-Hispanic White	High School/Some College	$75,000+	129	9114.88	1.09
Male	25–34	Non-Hispanic White	College Graduate	$20,000–$74,999	126	8810.29	1.06
Female	25–34	Non-Hispanic White	College Graduate	$20,000–$74,999	139	8724.57	1.05
Male	45–54	Non-Hispanic White	High School/Some College	$20,000–$74,999	284	8467.18	1.02
.	.	.	.	.	.	.	.
.	.	.	.	.	.	.	.
.	.	.	.	.	.	.	.
Total					36,525	833,990.70	100.00

**Table 3:** Number of non-consumers by animal protein and survey cycle.

		Protein	Beef	Pork	Poultry	Seafood	Combo	Egg	Dairy
Survey Cycle	Total *n*	*n* (Share)	*n* (Share)	*n* (Share)	*n* (Share)	*n* (Share)	*n* (Share)	*n* (Share)	*n* (Share)
2007–2008	5678	-	2513 (0.44)	3016 (0.53)	1964 (0.35)	4143 (0.73)	3500 (0.62)	3422 (0.60)	493 (0.09)
2009–2010	6046	-	2730 (0.45)	3322 (0.55)	2192 (0.36)	4278 (0.71)	3905 (0.65)	3622 (0.60)	503 (0.08)
2011–2012	5071	-	2616 (0.52)	2886 (0.57)	1678 (0.33)	3507 (0.69)	2740 (0.54)	3007 (0.59)	534 (0.11)
2013–2014	5354	-	2876 (0.54)	3042 (0.57)	1817 (0.34)	3742 (0.70)	2911 (0.54)	3077 (0.57)	572 (0.11)
2015–2016	5250	-	3047 (0.58)	3050 (0.58)	1883 (0.36)	3813 (0.73)	2851 (0.54)	2935 (0.56)	689 (0.13)
2017–2018	4976	-	2824 (0.57)	2956 (0.59)	1806 (0.36)	3575 (0.72)	2808 (0.56)	2792 (0.56)	649 (0.13)
Total	32,375	-	16,606 (0.51)	18,272 (0.56)	11,340 (0.35)	23,058 (0.71)	18,715 (0.58)	18,855 (0.58)	3440 (0.11)

**Table 4:** Daily protein consumption by NHANES survey cycle and participant characteristics.

(a)					
	Daily Consumption				
		Protein (g/d)	Beef (g/d)	Pork (g/d)	Poultry (g/d)
	*n*	Mean (SE)	Mean (SE)	Mean (SE)	Mean (SE)
Total	32,375	81.97 (0.29)	32.89 (0.42)	19.46 (0.30)	49.08 (0.49)
Survey Cycle					
2007–2008	5678	81.42 (0.69)	37.11 (0.99)	20.02 (0.73)	47.70 (1.09)
2009–2010	6046	83.13 (0.65)	37.75 (0.96)	21.29 (0.70)	49.94 (1.16)
2011–2012	5071	82.29 (0.73)	33.65 (1.11)	17.91 (0.68)	47.36 (1.11)
2013–2014	5354	83.28 (0.70)	31.09 (0.90)	19.32 (0.72)	49.99 (1.10)
2015–2016	5250	81.51 (0.76)	28.04 (1.13)	19.83 (0.80)	49.64 (1.36)
2017–2018	4976	80.29 (0.77)	30.39 (1.08)	18.48 (0.81)	49.72 (1.33)
Gender					
Male	15,785	96.26 (0.47)	43.12 (0.74)	25.16 (0.53)	56.61 (0.83)
Female	16,590	68.63 (0.28)	23.34 (0.41)	14.13 (0.31)	42.04 (0.54)
Age					
18–24	4225	84.23 (0.92)	33.71 (1.18)	17.09 (0.81)	58.11 (1.55)
25–34	5092	87.37 (0.76)	33.17 (0.98)	18.19 (0.69)	57.81 (1.29)
35–44	5130	86.20 (0.73)	35.06 (1.06)	19.52 (0.72)	53.40 (1.27)
45–54	5191	83.14 (0.69)	34.98 (1.10)	20.97 (0.78)	48.52 (1.12)
55–64	5400	80.55 (0.74)	32.46 (1.12)	20.60 (0.87)	44.06 (1.20)
65+	7337	71.39 (0.48)	28.34 (0.77)	19.73 (0.58)	35.39 (0.78)
Race/Ethnicity					
Non-Hispanic White	13,258	81.99 (0.40)	33.77 (0.58)	20.07 (0.42)	45.41 (0.66)
Non-Hispanic Black	7023	78.10 (0.55)	30.32 (0.78)	18.20 (0.52)	64.54 (1.04)
Mexican American	4988	86.86 (0.78)	36.35 (1.17)	16.13 (0.72)	50.44 (1.26)
Other	7106	81.93 (0.64)	28.66 (0.88)	19.72 (0.61)	52.78 (1.19)
Education *					
College Graduate	7115	85.80 (0.56)	28.62 (0.82)	16.82 (0.57)	51.57 (1.00)
High School/Some College	17,181	81.36 (0.41)	34.70 (0.58)	20.66 (0.42)	49.11 (0.67)
<High School	8019	77.36 (0.59)	34.10 (0.89)	19.94 (0.66)	44.54 (0.94)
Annual Household Income *					
<$20,000	6678	76.79 (0.67)	31.91 (0.91)	19.44 (0.73)	45.83 (1.05)
$20,000–$74,999	16,112	80.59 (0.41)	33.69 (0.58)	20.02 (0.43)	47.22 (0.64)
$75,000+	7880	86.27 (0.56)	32.47 (0.81)	19.01 (0.57)	52.64 (1.00)
Body Weight *					
50 kg or less	1009	71.53 (1.56)	24.81 (1.76)	16.90 (1.58)	41.05 (2.24)
50.01–65 kg	6228	73.19 (0.58)	25.13 (0.74)	15.31 (0.51)	42.86 (1.01)
65.01–80 kg	9959	79.96 (0.53)	30.86 (0.75)	18.46 (0.57)	47.18 (0.87)
80.01–95 kg	7720	85.64 (0.61)	35.40 (0.90)	20.39 (0.61)	52.32 (1.06)
>95 kg	7144	88.98 (0.64)	39.89 (0.99)	23.45 (0.73)	54.02 (1.09)
Physical Exercise					
0 min/d	16,851	77.68 (0.39)	33.77 (0.60)	20.55 (0.44)	44.98 (0.65)
1–30 min/d	8155	82.27 (0.53)	32.15 (0.82)	18.52 (0.59)	49.33 (0.92)
31–60 min/d	4261	85.88 (0.79)	30.60 (1.04)	18.45 (0.77)	53.86 (1.37)
61–120 min/d	2199	92.20 (1.32)	32.70 (1.66)	19.12 (1.11)	56.27 (1.98)
121–180 min/d	571	98.55 (3.40)	39.21 (3.00)	18.60 (1.95)	67.38 (6.21)
181–240 min/d	206	95.91 (4.01)	35.88 (6.17)	14.11 (2.32)	63.94 (6.58)
241–300 min/d	62	93.88 (4.85)	40.03 (10.05)	19.79 (6.38)	53.48 (7.85)
>300 min/d	70	91.67 (6.57)	46.21 (15.13)	19.08 (5.38)	60.3 (13.03)
**(b)**					
	**Daily Consumption**				
		**Seafood (g/d)**	**Combo (g/d)**	**Egg (g/d)**	**Dairy (c/d)**
	***n***	**Mean (SE)**	**Mean (SE)**	**Mean (SE)**	**Mean (SE)**
Total	32,375	17.64 (0.37)	14.53 (0.22)	12.37 (0.17)	1.22 (0.01)
Survey Cycle					
2007–2008	5678	16.01 (0.74)	12.62 (0.44)	10.91 (0.35)	1.29 (0.02)
2009–2010	6046	18.36 (0.94)	11.22 (0.41)	11.02 (0.33)	1.41 (0.02)
2011–2012	5071	18.01 (0.87)	16.19 (0.56)	11.67 (0.39)	1.25 (0.03)
2013–2014	5354	19.33 (0.83)	15.46 (0.49)	12.56 (0.38)	1.25 (0.03)
2015–2016	5250	17.18 (1.06)	16.22 (0.57)	13.58 (0.47)	1.12 (0.02)
2017–2018	4976	16.92 (0.87)	15.16 (0.66)	14.22 (0.50)	1.04 (0.03)
Gender					
Male	15,785	19.42 (0.60)	18.63 (0.36)	14.27 (0.27)	1.38 (0.02)
Female	16,590	15.98 (0.43)	10.70 (0.25)	10.60 (0.21)	1.08 (0.01)
Age					
18–24	4225	13.46 (0.88)	16.32 (0.58)	10.90 (0.46)	1.33 (0.03)
25–34	5092	15.79 (0.73)	16.43 (0.54)	13.44 (0.46)	1.26 (0.02)
35–44	5130	17.96 (0.99)	16.31 (0.55)	12.63 (0.42)	1.23 (0.03)
45–54	5191	18.57 (0.84)	14.99 (0.60)	12.28 (0.41)	1.22 (0.03)
55–64	5400	20.94 (1.18)	13.26 (0.56)	12.69 (0.43)	1.20 (0.03)
65+	7337	18.13 (0.67)	10.47 (0.37)	11.94 (0.30)	1.12 (0.02)
Race/Ethnicity					
Non-Hispanic White	13,258	15.63 (0.50)	14.49 (0.30)	11.62 (0.23)	1.35 (0.01)
Non-Hispanic Black	7023	22.54 (0.74)	15.09 (0.41)	12.34 (0.29)	0.85 (0.01)
Mexican American	4988	17.08 (0.98)	19.15 (0.65)	17.44 (0.49)	1.08 (0.02)
Other	7106	23.45 (0.78)	11.26 (0.39)	12.70 (0.36)	1.00 (0.02)
Education *					
College Graduate	7115	20.67 (0.68)	11.75 (0.40)	12.29 (0.34)	1.29 (0.02)
High School/Some College	17,181	16.48 (0.53)	15.65 (0.31)	12.14 (0.23)	1.22 (0.01)
<High School	8019	16.37 (0.66)	15.53 (0.45)	13.30 (0.34)	1.10 (0.02)
Annual Household Income *					
<$20,000	6678	14.56 (0.63)	15.06 (0.46)	12.30 (0.35)	1.19 (0.02)
$20,000–$74,999	16,112	16.90 (0.52)	15.10 (0.30)	12.51 (0.23)	1.20 (0.01)
$75,000+	7880	20.03 (0.73)	13.62 (0.43)	11.96 (0.32)	1.29 (0.02)
Body Weight *					
50 kg or less	1009	14.63 (1.40)	7.75 (0.67)	10.01 (0.75)	1.15 (0.06)
50.01–65 kg	6228	17.38 (0.70)	10.98 (0.40)	10.32 (0.32)	1.11 (0.02)
65.01–80 kg	9959	17.67 (0.68)	12.85 (0.37)	11.95 (0.30)	1.22 (0.02)
80.01–95 kg	7720	17.36 (0.67)	15.40 (0.41)	13.22 (0.37)	1.28 (0.02)
>95 kg	7144	18.63 (0.91)	19.26 (0.57)	13.85 (0.38)	1.26 (0.02)
Physical Exercise					
0 min/d	16,851	16.30 (0.56)	15.26 (0.30)	12.10 (0.23)	1.14 (0.01)
1–30 min/d	8155	17.26 (0.59)	14.11 (0.46)	11.41 (0.30)	1.25 (0.02)
31–60 min/d	4261	20.10 (0.90)	12.85 (0.53)	12.91 (0.47)	1.26 (0.03)
61–120 min/d	2199	20.44 (1.43)	14.16 (0.76)	15.06 (0.75)	1.46 (0.05)
121–180 min/d	571	23.43 (3.89)	15.21 (1.54)	15.24 (1.34)	1.51 (0.09)
181–240 min/d	206	20.04 (4.73)	19.56 (3.37)	17.30 (2.96)	1.45 (0.10)
241–300 min/d	62	10.16 (4.33)	25.15 (4.46)	14.74 (4.35)	1.38 (0.23)
>300 min/d	70	17.51 (12.32)	15.34 (3.85)	9.99 (2.66)	1.10 (0.19)

Note: * Across the entire period of study, 60, 1705, and 315 NHANES participants did not provide educational attainment, annual household income, and body weight, respectively. Observations for these traits will not match the total number of observations.

**Table 5:** OLS regression predicting total protein consumption.

Predictor	ME (g/d)	95% CI	*p*
Intercept	62.66	60.84, 64.48	<0.0001
Physical Exercise			
0 min/d	-	-	-
1–30 min/d	2.08	1.18, 2.98	<0.0001
31–60 min/d	3.24	2.14, 4.34	<0.0001
61–120 min/d	6.77	5.35, 8.20	<0.0001
121–180 min/d	12.41	9.72, 15.11	<0.0001
181–240 min/d	9.11	4.89, 13.32	<0.0001
241–300 min/d	7.57	−1.12, 16.26	0.088
>300 min/d	4.07	−4.43, 12.57	0.348
Survey Cycle			
2007–2008	-	-	-
2009–2010	1.28	0.01, 2.56	0.049
2011–2012	0.05	−1.22, 1.32	0.936
2013–2014	1.23	−0.03, 2.49	0.056
2015–2016	−0.50	−1.77, 0.76	0.435
2017–2018	−1.76	−3.02, −0.50	0.006
Gender			
Male	26.55	25.83, 27.28	<0.0001
Female	-	-	-
Age			
18–24	-	-	-
25–34	3.33	1.99, 4.67	<0.0001
35–44	2.67	1.31, 4.03	0.0001
45–54	0.19	−1.15, 1.54	0.777
55–64	−1.95	−3.32, −0.58	0.005
65+	−9.45	−10.81, −8.09	<0.0001
Race/Ethnicity			
Non-Hispanic White	-	-	-
Non-Hispanic Black	−2.61	−3.79, −1.43	<0.0001
Mexican American	4.97	3.61, 6.33	<0.0001
Other	−0.34	−1.43, 0.74	0.537
Education			
College Graduate	6.45	5.17, 7.72	<0.0001
High School/Some College	3.71	2.64, 4.78	<0.0001
<High School	-	-	-
Annual Household Income			
<$20,000	-	-	-
$20,000–$74,999	0.85	−0.25, 1.95	0.129
$75,000+	3.17	1.94, 4.39	<0.0001
Did Not Report	0.63	−1.39, 2.65	0.541
*n*	32,375		
Adj. R^2^	0.18		
Model F value	266.83		
Model *p* value	<0.0001		

**Table 6:** Multivariate Tobit conditional marginal effects on animal protein consumption.

(a)						
	Conditional Marginal Effect					
Predictor	Beef (g/d)	Pork (g/d)		Poultry (g/d)		Seafood (g/d)
Physical Exercise						
0 min/d	-	-		-		-
	-	-		-		-
1–30 min/d	−3.83	−4.31 *		9.21 *		9.41 *
	[−8.09, 0.43]	[−7.59, −1.03]		[5.11, 13.31]		[3.76, 15.06]
31–60 min/d	−13.30 *	−8.31 *		13.87 *		26.46 *
	[−18.49, −8.11]	[−12.38, −4.23]		[8.88, 18.86]		[19.57, 33.34]
61–120 min/d	−16.13 *	−7.53 *		10.60 *		23.25 *
	[−22.89, −9.37]	[−12.74, −2.32]		[4.12, 17.09]		[14.20, 32.30]
121–180 min/d	7.56	−8.52		21.93 *		18.76 *
	[−4.98, 20.10]	[−18.40, 1.37]		[9.69, 34.17]		[1.33, 36.20]
181–240 min/d	−7.74	−19.30 *		27.88 *		10.17
	[−27.55, 12.07]	[−34.62, −3.99]		[8.87, 46.89]		[−17.2, 37.55]
241–300 min/d	2.31	−11.10		−4.88		−16.59
	[−37.95, 42.58]	[−42.90, 20.70]		[−44.46, 34.70]		[−75.82, 42.64]
>300 min/d	9.46	−10.50		7.12		2.02
	[−30.12, 49.05]	[−41.84, 20.85]		[−30.72, 44.96]		[−54.29, 58.34]
Survey Cycle						
2007–2008	-	-		-		-
	-	-		-		-
2009–2010	−0.23	1.44		1.98		8.97 *
	[−6.14, 5.69]	[−3.18, 6.05]		[−3.74, 7.71]		[0.97, 16.97]
2011–2012	−11.17 *	−9.37 *		−1.86		2.90
	[−17.06, −5.27]	[−13.98, −4.75]		[−7.55, 3.83]		[−5.09, 10.88]
2013–2014	−20.04 *	−3.97		3.14		6.40
	[−25.84, −14.24]	[−8.59, 0.65]		[−2.54, 8.82]		[−1.51, 14.32]
2015–2016	−34.24 *	−4.16		−1.50		−16.61 *
	[−40.13, −28.35]	[−8.77, 0.45]		[−7.21, 4.21]		[−24.63, −8.60]
2017–2018	−22.98 *	−7.48 *		0.82		−10.02 *
	[−28.83, −17.13]	[−12.02, −2.94]		[−4.91, 6.55]		[−17.95, −2.09]
Gender						
Male	52.58 *	32.34 *		23.81 *		−3.40
	[49.13, 56.04]	[29.65, 35.02]		[20.51, 27.10]		[−7.92, 1.12]
Female	-	-		-		-
	-	-		-		-
Age						
18–24	-	-		-		-
	-	-		-		-
25–34	4.44	10.62 *		1.30		12.26 *
	[−1.97, 10.84]	[5.44, 15.81]		[−4.79, 7.39]		[3.32, 21.20]
35–44	12.72 *	17.20 *		−6.03		25.62 *
	[6.30, 19.15]	[11.98, 22.42]		[−12.13, 0.08]		[16.55, 34.69]
45–54	10.67 *	21.73 *		−17.81 *		36.01 *
	[4.29, 17.04]	[16.62, 26.83]		[−23.94, −11.69]		[26.95, 45.08]
55–64	6.26	23.55 *		−26.04 *		53.71 *
	[−0.26, 12.77]	[18.25, 28.84]		[−32.27, −19.81]		[44.41, 63.01]
65+	−4.29	25.31 *		−40.79 *		57.49 *
	[−10.76, 2.18]	[20.14, 30.48]		[−46.87, −34.70]		[48.27, 66.70]
Race/Ethnicity						
Non-Hispanic White	−	−		−		−
	−	−		−		−
Non-Hispanic Black	−12.43 *	−1.82		49.06 *		52.75 *
	[−18.07, −6.79]	[−6.08, 2.45]		[43.71, 54.41]		[45.25, 60.25]
Mexican American	−0.01	−17.87 *		12.97 *		31.57 *
	[−6.39, 6.37]	[−22.84, −12.90]		[6.77, 19.17]		[22.69, 40.45]
Other	−18.33 *	−3.00		14.80 *		54.82 *
	[−23.52, −13.14]	[−6.99, 1.00]		[9.85, 19.75]		[47.98, 61.67]
Education						
College Graduate	−13.49 *	−12.97 *		20.30 *		31.90 *
	[−19.51, −7.47]	[−17.62, −8.32]		[14.43, 26.17]		[23.86, 39.95]
High School/Some College	6.16 *	3.96 *		13.21 *		7.20 *
	[1.13, 11.20]	[0.03, 7.88]		[8.23, 18.18]		[0.43, 13.97]
<High School	-	-		-		-
	-	-		-		-
Annual Household Income						
<$20,000	-	-		-		-
	-	-		-		-
$20,000–$74,999	3.71	3.09		5.86 *		17.48 *
	[−1.43, 8.84]	[−0.93, 7.11]		[0.86, 10.87]		[10.28, 24.67]
$75,000+	0.55	0.98		16.37 *		35.32 *
	[−5.20, 6.29]	[−3.52, 5.48]		[10.78, 21.96]		[27.26, 43.38]
Did Not Report	−5.34	−8.88 *		11.97 *		21.08 *
	[−14.85, 4.17]	[−16.26, −1.49]		[2.87, 21.07]		[8.19, 33.97]
*n*	32,375					
Log-Likelihood	−605,669					
AIC	1,211,772					
**(b)**						
	**Conditional Marginal Effect**					
**Predictor**	**Combo (g/d)**		**Egg (g/d)**		**Dairy (c/d)**	
Physical Exercise						
0 min/d	-		-		-	
	-		-		-	
1–30 min/d	−3.10 *		−0.28		0.15 *	
	[−5.39, −0.81]		[−2.32, 1.76]		[0.10, 0.21]	
31–60 min/d	−10.05 *		3.88 *		0.09 *	
	[−12.81, −7.29]		[1.36, 6.39]		[0.02, 0.16]	
61–120 min/d	−11.32 *		8.71 *		0.40 *	
	[−14.90, −7.74]		[5.47, 11.96]		[0.31, 0.49]	
121–180 min/d	−9.67 *		9.41 *		0.60 *	
	[−16.29, −3.04]		[3.19, 15.63]		[0.43, 0.78]	
181–240 min/d	0.95		13.01 *		0.45 *	
	[−9.39, 11.29]		[3.37, 22.64]		[0.18, 0.72]	
241–300 min/d	18.15		7.07		0.42	
	[−2.70, 39.00]		[−12.56, 26.70]		[−0.14, 0.97]	
>300 min/d	−15.91		−9.81		−0.25	
	[−36.94, 5.13]		[−29.57, 9.94]		[−0.79, 0.30]	
Survey Cycle						
2007–2008	-		-		-	
	-		-		-	
2009–2010	−5.48 *		0.33		0.24 *	
	[−8.75, −2.21]		[−2.57, 3.24]		[0.15, 0.32]	
2011–2012	15.64 *		2.51		−0.06	
	[12.33, 18.95]		[−0.41, 5.43]		[−0.14, 0.02]	
2013–2014	11.17 *		6.13 *		−0.09 *	
	[7.90, 14.44]		[3.23, 9.04]		[−0.17, −0.01]	
2015–2016	16.19 *		7.44 *		−0.30 *	
	[12.90, 19.48]		[4.53, 10.34]		[−0.38, −0.22]	
2017–2018	13.18 *		9.07 *		−0.41 *	
	[9.92, 16.44]		[6.15, 12.00]		[−0.49, −0.33]	
Gender						
Male	23.28 *		7.72 *		0.44 *	
	[21.41, 25.14]		[6.05, 9.39]		[0.40, 0.49]	
Female	-		-		-	
	-		-		-	
Age						
18–24	-		-		-	
	-		-		-	
25–34	1.00		11.95 *		−0.12 *	
	[−2.36, 4.35]		[8.75, 15.15]		[−0.21, −0.04]	
35–44	−0.18		12.68 *		−0.16 *	
	[−3.56, 3.21]		[9.45, 15.92]		[−0.25, −0.08]	
45–54	−5.10 *		13.91 *		−0.20 *	
	[−8.46, −1.74]		[10.69, 17.14]		[−0.28, −0.11]	
55–64	−13.01 *		17.07 *		−0.26 *	
	[−16.36, −9.66]		[13.78, 20.36]		[−0.35, −0.18]	
65+	−22.54 *		16.91 *		−0.36 *	
	[−25.92, −19.16]		[13.67, 20.15]		[−0.45, −0.28]	
Race/Ethnicity						
Non-Hispanic White	-		-		-	
	-		-		-	
Non-Hispanic Black	−4.19 *		5.58 *		−0.94 *	
	[−7.12, −1.25]		[2.90, 8.25]		[−1.02, −0.87]	
Mexican American	3.81 *		19.84 *		−0.51 *	
	[0.45, 7.17]		[16.75, 22.93]		[−0.59, −0.42]	
Other	−17.50 *		4.46 *		−0.67 *	
	[−20.29, −14.72]		[1.95, 6.97]		[−0.74, −0.60]	
Education						
College Graduate	−8.27 *		0.81		0.21 *	
	[−11.51, −5.02]		[−2.05, 3.66]		[0.13, 0.29]	
High School/Some College	2.71		−0.86		0.10 *	
	[−0.01, 5.44]		[−3.27, 1.56]		[0.03, 0.17]	
<High School	-		-		-	
	-		-		-	
Annual Household Income						
<$20,000	-		-		-	
	-		-		-	
$20,000–$74,999	1.08		1.41		−0.08 *	
	[−1.73, 3.89]		[−1.11, 3.93]		[−0.15, 0.00]	
$75,000+	−2.40		−1.05		−0.05	
	[−5.51, 0.72]		[−3.84, 1.74]		[−0.13, 0.03]	
Did Not Report	−3.89		2.98		−0.15 *	
	[−9.04, 1.26]		[−1.52, 7.48]		[−0.28, −0.02]	
*n*	32,375					
Log-Likelihood	−605,669					
AIC	1,211,772					

Note: Asterisks (*) indicate a marginal effect that is statistically significant at the 95 percent confidence level. Values in brackets [ ] are 95 percent confidence intervals around the marginal effect, calculated using Krinsky–Robb standard errors. Log-likelihood and AIC values are from the original multivariate Tobit estimation.

## Data Availability

Publicly available data used in this study is available at https://wwwn.cdc.gov/nchs/nhanes/Default.aspx, accessed on 10 April 2024.

## Appendix A

**Table A1:** Multivariate tobit unconditional marginal effects on animal protein consumption.

(a)						
	Unconditional Marginal Effect					
Predictor	Beef (g/d)	Pork (g/d)		Poultry (g/d)		Seafood (g/d)
Physical Exercise						
0 min/d	-	-		-		-
	-	-		-		-
1–30 min/d	−1.31	−2.05 *		0.36 *		7.00 *
	[−2.80, 0.17]	[−3.64, −0.46]		[0.21, 0.51]		[2.85, 11.16]
31–60 min/d	−4.86 *	−4.07 *		0.38 *		18.86 *
	[−6.90, −2.82]	[−6.17, −1.98]		[0.26, 0.50]		[14.21, 23.52]
61–120 min/d	−6.08 *	−3.70 *		0.32 *		16.55 *
	[−8.91, −3.26]	[−6.40, −1.01]		[0.19, 0.44]		[10.48, 22.62]
121–180 min/d	2.40	−4.24		0.14		13.44 *
	[−1.35, 6.16]	[−9.47, 0.98]		[−0.32, 0.60]		[1.61, 25.27]
181–240 min/d	−2.78	−10.40 *		−0.14		7.46
	[−10.33, 4.78]	[−19.70, −1.09]		[−1.24, 0.96]		[−12.02, 26.93]
241–300 min/d	0.77	−5.65		−0.29		−13.05
	[−12.62, 14.16]	[−23.13, 11.83]		[−4.17, 3.60]		[−60.83, 34.72]
>300 min/d	2.95	−5.32		0.24		1.52
	[−8.67, 14.56]	[−22.32, 11.69]		[−2.03, 2.51]		[−40.06, 43.10]
Survey Cycle						
2007–2008	-	-		-		-
	-	-		-		-
2009–2010	−0.08	0.67		0.09		6.66 *
	[−2.08, 1.93]	[−1.46, 2.79]		[−0.16, 0.35]		[0.81, 12.50]
2011–2012	−4.01 *	−4.60 *		−0.10		2.17
	[−6.26, −1.77]	[−6.98, −2.23]		[−0.41, 0.21]		[−3.78, 8.13]
2013–2014	−7.52 *	−1.90		0.14		4.77
	[−9.93, −5.12]	[−4.15, 0.35]		[−0.09, 0.37]		[−1.06, 10.60]
2015–2016	−13.80 *	−1.99		−0.08		−12.90 *
	[−16.59, −11.02]	[−4.24, 0.26]		[−0.38, 0.23]		[−19.29, −6.50]
2017–2018	−8.79 *	−3.64 *		0.04		−7.69 *
	[−11.30, −6.29]	[−5.93, −1.35]		[−0.23, 0.31]		[−13.89, −1.50]
Gender						
Male	17.55 *	14.99 *		1.14 *		−2.56
	[16.25, 18.85]	[13.69, 16.29]		[0.90, 1.39]		[−5.98, 0.85]
Female	-	-		-		-
	-	-		-		-
Age						
18–24	-	-		-		-
	-	-		-		-
25–34	1.46	4.68 *		0.06		9.03 *
	[−0.60, 3.52]	[2.53, 6.83]		[−0.22, 0.34]		[2.59, 15.46]
35–44	3.99 *	7.29 *		−0.35		18.38 *
	[2.12, 5.86]	[5.29, 9.30]		[−0.76, 0.07]		[12.19, 24.57]
45–54	3.39 *	8.97 *		−1.35 *		25.28 *
	[1.48, 5.29]	[7.12, 10.83]		[−2.00, −0.69]		[19.35, 31.21]
55–64	2.04	9.64 *		−2.29 *		36.34 *
	[−0.01, 4.09]	[7.76, 11.53]		[−3.14, −1.44]		[30.71, 41.97]
65+	−1.48	10.53 *		−4.09 *		39.39 *
	[−3.75, 0.80]	[8.64, 12.43]		[−5.15, −3.02]		[33.67, 45.11]
Race/Ethnicity						
Non-Hispanic White	-	-		-		-
	-	-		-		-
Non-Hispanic Black	−4.44 *	−0.86		−0.17		36.35 *
	[−6.62, −2.26]	[−2.89, 1.18]		[−0.67, 0.33]		[31.77, 40.93]
Mexican American	0.00	−9.18 *		0.39 *		22.35 *
	[−2.16, 2.16]	[−12.01, −6.34]		[0.27, 0.52]		[16.54, 28.15]
Other	−6.72 *	−1.42		0.47 *		37.66 *
	[−8.85, −4.60]	[−3.35, 0.51]		[0.34, 0.59]		[33.46, 41.87]
Education						
College Graduate	−4.84 *	−6.42 *		0.52 *		22.84 *
	[−7.13, −2.56]	[−8.84, −3.99]		[0.35, 0.69]		[17.33, 28.36]
High School/Some College	2.09 *	1.85 *		0.67 *		5.43 *
	[0.37, 3.81]	[0.00, 3.70]		[0.38, 0.96]		[0.31, 10.56]
<High School	-	-		-		-
	-	-		-		-
Annual Household Income						
<$20,000	-	-		-		-
	-	-		-		-
$20,000–$74,999	1.25	1.45		0.29 *		13.17 *
	[−0.48, 2.99]	[−0.44, 3.33]		[0.03, 0.54]		[7.73, 18.61]
$75,000+	0.18	0.46		0.52 *		25.26 *
	[−1.75, 2.12]	[−1.63, 2.54]		[0.35, 0.68]		[19.74, 30.78]
Did Not Report	−1.87	−4.41 *		0.32 *		15.07 *
	[−5.34, 1.59]	[−8.31, −0.52]		[0.17, 0.47]		[6.35, 23.78]
*n*	32,375					
Log-Likelihood	−605,669					
AIC	1,211,772					
**(b)**						
	**Unconditional Marginal Effect**					
**Predictor**	**Combo (g/d)**		**Egg (g/d)**		**Dairy (c/d)**	
Physical Exercise						
0 min/d	-		-		-	
	-		-		-	
1–30 min/d	−1.48 *		−0.13		−0.03 *	
	[−2.59, −0.37]		[−1.08, 0.82]		[−0.04, −0.02]	
31–60 min/d	−5.10 *		1.73 *		−0.01 *	
	[−6.62, −3.59]		[0.65, 2.81]		[−0.03, 0.00]	
61–120 min/d	−5.89 *		3.64 *		−0.07 *	
	[−7.95, −3.83]		[2.42, 4.86]		[−0.09, −0.05]	
121–180 min/d	−5.01 *		3.85 *		−0.11 *	
	[−8.77, −1.24]		[1.64, 6.06]		[−0.14, −0.08]	
181–240 min/d	0.44		5.03 *		−0.08 *	
	[−4.31, 5.19]		[2.01, 8.04]		[−0.13, −0.03]	
241–300 min/d	6.77 *		2.98		−0.07	
	[0.75, 12.79]		[−4.54, 10.50]		[−0.18, 0.03]	
>300 min/d	−8.79		−5.13		0.04	
	[−22.05, 4.46]		[−16.56, 6.30]		[−0.03, 0.11]	
Survey Cycle						
2007–2008	-		-		-	
	-		-		-	
2009–2010	−2.67 *		0.15		−0.04 *	
	[−4.32, −1.01]		[−1.18, 1.49]		[−0.05, −0.03]	
2011–2012	6.43 *		1.14		0.01	
	[5.22, 7.63]		[−0.16, 2.43]		[0.00, 0.02]	
2013–2014	4.79 *		2.69 *		0.01 *	
	[3.49, 6.08]		[1.48, 3.90]		[0.00, 0.03]	
2015–2016	6.64 *		3.22 *		0.05 *	
	[5.44, 7.83]		[2.04, 4.40]		[0.03, 0.06]	
2017–2018	5.53 *		3.86 *		0.06 *	
	[4.29, 6.77]		[2.71, 5.01]		[0.05, 0.07]	
Gender						
Male	10.80 *		3.57 *		−0.07 *	
	[9.89, 11.71]		[2.79, 4.34]		[−0.08, −0.06]	
Female	-		-		-	
	-		-		-	
Age						
18–24	-		-		-	
	-		-		-	
25–34	0.46		4.95 *		0.02 *	
	[−1.08, 2.01]		[3.76, 6.13]		[0.01, 0.03]	
35–44	−0.08		5.21 *		0.03 *	
	[−1.67, 1.51]		[4.04, 6.39]		[0.01, 0.04]	
45–54	−2.48 *		5.65 *		0.03 *	
	[−4.18, −0.78]		[4.51, 6.80]		[0.02, 0.04]	
55–64	−6.70 *		6.74 *		0.04 *	
	[−8.59, −4.80]		[5.64, 7.84]		[0.03, 0.05]	
65+	−12.01 *		6.89 *		0.06 *	
	[−14.09, −9.94]		[5.74, 8.04]		[0.04, 0.07]	
Race/Ethnicity						
Non-Hispanic White	-		-		-	
	-		-		-	
Non-Hispanic Black	−2.02 *		2.48 *		0.12 *	
	[−3.48, −0.55]		[1.36, 3.60]		[0.11, 0.12]	
Mexican American	1.73 *		7.56 *		0.07 *	
	[0.26, 3.20]		[6.65, 8.47]		[0.06, 0.08]	
Other	−9.11 *		2.00 *		0.09 *	
	[−10.78, −7.43]		[0.93, 3.08]		[0.09, 0.10]	
Education						
College Graduate	−4.08 *		0.37		−0.04 *	
	[−5.75, −2.40]		[−0.94, 1.68]		[−0.05, −0.02]	
High School/Some College	1.27		−0.40		−0.02 *	
	[−0.01, 2.56]		[−1.52, 0.72]		[−0.03, −0.01]	
<High School	-		-		-	
	-		-		-	
Annual Household Income						
<$20,000	-		-		-	
	-		-		-	
$20,000–$74,999	0.51		0.65		0.01 *	
	[−0.81, 1.82]		[−0.51, 1.82]		[0.00, 0.02]	
$75,000+	−1.14		−0.49		0.01	
	[−2.64, 0.36]		[−1.80, 0.82]		[0.00, 0.02]	
Did Not Report	−1.89		1.33		0.02 *	
	[−4.50, 0.71]		[−0.61, 3.28]		[0.00, 0.04]	
*n*	32,375					
Log-Likelihood	−605,669					
AIC	1,211,772					

Note: Asterisks (*) indicate a marginal effect that is statistically significant at the 95 percent confidence level. Values in brackets [ ] are 95 percent confidence intervals around the marginal effect, calculated using Krinsky-Robb standard errors. Log-likelihood and AIC values are from the original multivariate Tobit estimation.
